# Betahistine in the treatment of tinnitus in patients with vestibular disorders

**DOI:** 10.1590/S1808-86942011000400014

**Published:** 2015-10-19

**Authors:** Maurício Malavasi Ganança, Heloisa Helena Caovilla, Juliana Maria Gazzola, Cristina Freitas Ganança, Fernando Freitas Ganança

**Affiliations:** 1 Full Professor of Otorhinolaryngology - Federal University of São Paulo (UNIFESP) - Paulista School of Medicine (EPM). Professor at the Professional Master's Degree Program in Body Balance Rehabilitation and Social Inclusion at the Bandeirante University of São Paulo; 2 Senior Associate Professor of Neurotology. Associate Professor - Otology and Neurotology Program - Federal University of São Paulo - Paulista School of Medicine; 3 PhD in Sciences - Graduate Program in Otorhinolaryngology and Head and Neck Surgery - Federal University of São Paulo - Paulista School of Medicine. Professor at the Professional Master's Degree Program in Body Balance Rehabilitation and Social Inclusion at the Bandeirante University of São Paulo; 4 PhD in Sciences - Human Communication Disorders Graduate Program - Department of Speech and Hearing Therapy - Federal University of São Paulo - Paulista School of Medicine; 5 Otorhinolaryngologist; Post-Doctorate from UNIFESP - EPM. Adjunct Professor; Head of the Otology and Neurotology Program - UNIFESP - EPM. Universidade Federal de São Paulo

**Keywords:** dizziness, medication therapy management, tinnitus, vertigo

## Abstract

**Abstract:**

Betahistine is a medicine used to treat vestibular disorders that has also been used to treat tinnitus.

**Aim:**

To assess the effects of betahistine on tinnitus in patients with vestibular disorders.

**Material and method:**

Retrospective data were collected from patient records for individuals presenting with vestibular dysfunction and tinnitus. Patients included had received betahistine 48 mg/day and clinical outcomes were compared with a control group comprising individuals who were unable to receive betahistine due to gastritis, ulcers, pregnancy, asthma or hypersensitivity to the drug. Patients underwent control of any aggravating factors and also standard vestibular exercises as a basis for treatment. The intensity, frequency and duration of tinnitus were assessed on the first day of dosing and after 120 days of treatment. Clinical improvement was defined as a total or partial reduction of tinnitus after treatment.

**Results:**

Clinical improvement was observed in 80/262 (30.5%) of patients treated with betahistine and 43/252 (17.1%) of control patients. Betahistine significantly (*p*<0.0001) improved tinnitus in treated individuals.

**Conclusion:**

The daily dosage of 48 mg of betahistine during 120 consecutive days is useful to reduce or eliminate tinnitus in patients with vestibular disorders.

## INTRODUCTION

Tinnitus, the perception of a sound in the absence of an apparent acoustic stimulus, is a growing health concern in all layers of the population. Estimates indicate that about 10%-15% of the population may have tinnitus. Adults and children may be affected, the prevalence of tinnitus increases with age and there is a high incidence associated with noise exposure such as age-related hearing loss. Although we still need to better understand its pathophysiology, tinnitus could be the result of a spontaneous and aberrant neural activity at any level of the hearing system^1^.

Approximately 8% of individuals with tinnitus are severely handicapped by this anguishing symptom; in 2,800 patients with tinnitus there was a significant association (*p*<0.05) between the symptom and the reduction in quality of life^2^. There is no influence from gender, age and hearing loss concerning the tinnitus nuisanse^3^.

Subjective tinnitus is a common symptom, with many causes^4^. One epidemiologic study showed that the most common cause for tinnitus was noise-induced hearing loss (37.8% of the cases); notwithstanding, many cases may be associated with other disorders, such as pain, stress, depression and headaches caused by migraine^5^. Ear disorders, especially the ones happening together with conductive or sensorineural hearing loss, such as excessive exposure to intense noise, presbycusis, metabolic disorders, ototoxic agents, Ménière's disease, vestibular schwannoma and psychological disorders are among the most frequent causes^6^. Tinnitus may also happen in patients with normal audiometry; normal-hearing patients with tinnitus have clinical characteristics which are similar to those of patients with hearing loss; however, the age range affected and the impact on concentration and emotional balance were significantly lower^7^. In elderly patients, in whom the main finding upon audiometry was presbycusis, no correlation was found between the degree of hearing loss and the extent to which the patient was unhappy with the tinnitus^8^. Tinnitus is one of the classical symptoms of Ménière's disease, a condition associated with substantial loss in quality of life^9^.

Tinnitus treatment is as diversified as the origin of the condition itself. Apparent causes must be treated. Controlling the cause may not be enough to reduce or even eliminate the tinnitus. With varying degrees of success, different treatments have been proposed for the tinnitus, such as tinnitus retraining therapy, sound masking, hearing aid fitting, acupuncture and drug therapy.

Numerous pharmaceutical options have been investigated^10^, ^11^, ^12^, with different degrees of quality of life improvement and symptom relief^1^^-^^2,^^13^. Drugs tried were: membrane stabilizers (lidocaine chlorhydrate), anti-seizure agents (carbamazepine, gabapentin), antidepressants (fluoxetine chlorhydrate; sertraline chlorhydrate), anxiolytics (clonazepam, alprazolam), Ginkgo biloba 761 extract, vasomodulators (flunarizine, cinnarizine and betahistine); the time of use for each drug and the criterion of choice depends on the clinical characteristics of each patient^14^.

There are studies showing that betahistine may provide symptom relief for tinnitus^15^, ^16^, ^17^, ^18^. Betahistine is an H3 antagonist bloodreceptor and an H1 agonist receptor which improves inner ear microcirculation^19^, promoting and facilitating central vestibular compensation^20^, ^21^, ^22^. It is currently recommended to treat symptoms from many vestibular disorders^23^^-^^24^, including tinnitus. Possible adverse events arising from betahistine use include headache, and epigastric discomfort. Betahistine is contraindicated in patients with gastrointestinal ulcers, asthma, pheochromocytoma and hypersensitivity to the drug^23^.

The goal of the present study was to assess the effects of betahistine on tinnitus in patients with vestibular dysfunction.

## METHODS

This was a case-controlled, retrospective and experimental study, carried out in a university facility. We retrospectively collected data from 865 patients with ages above 18 years who had tinnitus and peripheral vestibular dysfunction. The study was approved by the Ethics in Research Committee of the University where it was carried out, under protocol # 0117/08.

Patients were submitted to a neurotological evaluation which included clinical history; ear, nose and throat exam, audiological and vestibular assessments. Hearing was assessed by means of the tonal audiometry, speech recognition and acoustic immittance tests. Auditory processing, brainstem audiometry and/or electrocochleography were eventually carried out. Body balance assessment included the Romberg and Unterberger-Fukuda tests, positional and positioning nystagmus and electrocochleography.

We included patients with a diagnosis of peripheral vestibular disorders. We took off the study those patients with central nervous system disorders and those who did not conclude the treatment, or those who used other anti-dizziness medication or tinnitus-causing drugs. The patients who could use betahistine made up the study group, while the control group had those patients who could not use the drug for numerous reasons, including severe gastritis, ulcer, pregnancy, asthma and hypersensitivity to the drug.

The study group patients received 48 mg/day of betahistine dichlorohydrate BID or TID during 120 days. All patients controlled worsening factors and did vestibular rehabilitation exercises as base treatment. With the goal of homogeneously comparing the clinical results between control and treatment groups, a random sample of patients from the study group was chosen in order to have a similar number of individuals in each group for statistical purposes.

The patients were observed in the beginning and four months into therapy. The patient's overall impression was used as a variable to assess treatment efficacy. Effectiveness was assessed according to the patient's subjective response at the end of treatment. Efficacy assessment was done by means of the following classification: 1 = complete improvement (symptom-free patient), 2 = very good improvement, 3 = good improvement, 4 = mild improvement and 5 = no improvement. Patients with very good improvement, good improvement and mild improvement were included in the improvement class (partial improvement). Tolerability was judged by the investigators and the patient at the end of treatment. The chi-squared test was utilized in order to statistically compare tinnitus clinical improvement between the treatment and control groups. The significance level was 5% (α= 0.05). Statistical analyses were carried out using the SPSS 10.0 for Windows (Statistical Package for Social Sciences, version 10.0, 1999).

## RESULTS

**Figure 1:**
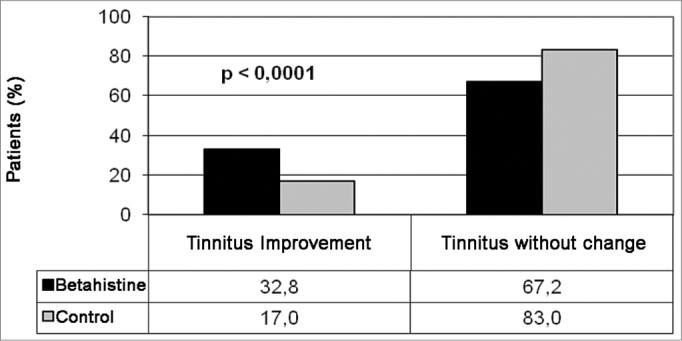
Comparing tinnitus clinical improvement among patients from the study and the control groups.

**Figure 2:**
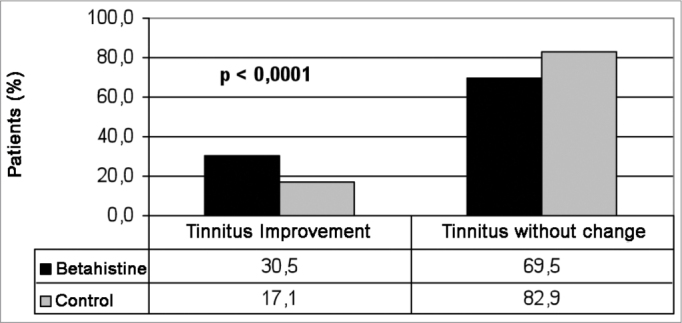
Comparing tinnitus clinical improvement between a random sample from the study and the control groups.

We had a total of 613 (70.9%) patients in the study group and 252 (29.1) in the control group. Clinical improvement was seen in 202 (32.8%) patients in the study group and in 43 (17%) patients in the control group, as per depicted in Figure 1. In order to compare the clinical results of tinnitus between the groups, a random sample of 42.7% patients in the study group (n=262) was selected in order to provide for a similar number of individuals from the study and from control for analysis purposes (Figure 2). A total of 514 patients made up the series in the present study. The experiment group was made up of 262 patients (51.0%), with ages varying between 19 and 76 years (mean value of 54.5 years); 160 females (61.1%) and 102 (39.9%) males; and the control group was made up of 252 patients (49.0%), with ages varying between 22 and 79 years (mean of 56.0 years); 156 females (62.4%) and 90 (37.6%) males. We did not notice any difference between the study and control groups as far as the number of treated cases was concerned, nor in regards to age and gender. We noticed clinical improvement in 80/262 (30.5%) patients treated with betahistine and in 43/252 (17.1%) patients in the control group (Table 1). There was a statistically significant difference (*p*<0.0001) between results from the control and study groups as far as the number of patients who improved is concerned, thus showing betahistine efficacy in symptom remission or tinnitus cure in patients with vestibular dysfunction.

**Table 1:** Number of patients per response class

Response class	Betahistine	Control
1 = complete improvement	18	3
2 = very good improvement	21	8
3 = good improvement	26	12
4 = mild improvement	15	20
5 = no improvement	182	209
	262	252

Treatment with betahistine was well tolerated, with low incidence of mild and transitional adverse effects, such as headaches, epigastric discomfort and dyspepsia. It was not necessary to break the use of the drug in any of the patients who suffered adverse effects.

## DISCUSSION

Betahistine is currently utilized in the treatment of numerous vestibular disorders^24^. In a review about the effects of betahistine in patients with Ménière's disease, some studies showed results which were considered satisfactory to reduce vertigo and tinnitus; and one study showed that betahistine did not have any effect on tinnitus when compared to placebo^15^. More recent clinical studies have shown that betahistine is efficient in the treatment of Ménière's disease symptoms^9,^^25^^-^^26^. Findings from the present study have also shown betahistine activity in reducing tinnitus in patients with vestibular disorders. A significant clinical improvement (*p*<0.0001) was seen in 30.5% of the patients treated with betahistine, when compared to 17.1% of patients who improved in the control group.

Other studies have also reported tinnitus improvement with the use of betahistine. In Ménière's disease patients there was tinnitus reduction with betahistine dimesylate (36 mg/day), as well as with a combination of 20 mg of cinnarizine and 40 mg of dimenhydrinate, and there was not statistically significant difference between the two treatment groups^27^. In another study, the five-week use of betahistine mesylate, in combination with B complex vitamin and diazepam significantly reduced tinnitus^28^. It is not possible to compare these findings, since the present investigation was carried out with betahistine dichlorohydrate and with different formulation and dosage.

Betahistine was not directly compared to other pharmacological approaches to treat tinnitus, thus it is difficult to estimate its efficacy when compared to other alternatives. Antidepressants are usually prescribed to treat tinnitus. There are reports regarding the use of fluoxetine and paroxetine; retrospective reviews with imipramine and selective inhibitors of serotonin reuptake; studies with amitriptyline and double-blind studies with trimipramine, nortriptyline, paroxetine and sertraline, compared to placebo. On the other hand, the literature also mentions that tinnitus may be an adverse effect of antidepressant drugs, such as phenelzine, amitriptyline, protriptyline, doxepin, imipramine, fluoxetine, trazodone, bupropion, venlafaxine. Tinnitus may also be associated with the interruption of antidepressant drugs such as venlafaxine and sertralina^29^, ^30^, ^31^.

The evidence concerning the pharmacological approaches to treat tinnitus is being built. The data from the present study suggests that betahistine can be a useful and well tolerated treatment option to be considered. We did not find other studies comparing patients with tinnitus and peripheral vestibular syndromes. We still need controlled prospective studies in order to confirm the present results.

## CONCLUSION

The 48 mg/day dose of betahistine during 120 consecutive days is useful to reduce or eliminate tinnitus in patients with vestibular disorders.
